# Association between serum vitamin E and bacterial vaginitis in women: a cross-sectional study

**DOI:** 10.1186/s12905-024-03065-4

**Published:** 2024-06-01

**Authors:** En-Hui Liu, Wan-Zhe Liao, Hao-Kai Chen, Xiao-Ye Huang, Rui-Xuan Li, Hao-Wen Liang, Xu-Guang Guo

**Affiliations:** 1 Department of Clinical Laboratory Medicine, Guangdong Provincial Key Laboratory of Major Obstetric Diseases; Guangdong Provincial Clinical Research Center for Obstetrics and Gynecology; The Third Affiliated Hospital of Guangzhou Medical University, Guangzhou, 510150 China; 2 Department of Pediatrics, Pediatrics School, Guangzhou Medical University, Guangzhou, 511436 China; 3 Department of Clinical Medicine, The Nanshan College of Guangzhou Medical University, Guangzhou, 511436 China; 4 Department of Clinical Medicine, The Third Clinical School of Guangzhou Medical University, Guangzhou, 511436 China; 5 Department of Public Utilities Management, The Health Management College of Guangzhou Medical University, Guangzhou, 511436 China; 6 Guangzhou Key Laboratory for Clinical Rapid Diagnosis and Early Warning of Infectious Diseases, King Med School of Laboratory Medicine, Guangzhou Medical University, Guangzhou, 510000 China

**Keywords:** Vitamin E, Bacterial vaginitis, National Health and Nutrition Examination Survey, Female diseases, Cross-sectional study

## Abstract

**Introduction:**

Bacterial vaginitis (BV) is a common vaginal disease. Vitamin E has been shown to reduce BV by enhancing immune function, but no studies have analyzed the relationship between vitamin E and BV at different BMIs and ages.

**Method:**

This study used 2242 participants from four cycles of NHANES 1999–2006 in American. Participants' vitamin E levels were divided into four groups, and analyses such as study population description, stratified analysis, multiple logistic regression analysis, and curve fitting were performed. To perform data processing, the researchers used the statistical package R (The R Foundation; http://www.r-project.org; version 3.6.3) and Empower Stats software (www.empowerstats.net, X&Y solutions, Inc. Boston, Massachusetts).

**Result:**

The concentrations of serum vitamin E were negatively correlated with the risk of BV, especially when vitamin E were at 1198-5459ug/dL with (OR = -0.443, 95%CI = 0.447–0.923, *P* = 0.032) or without (OR = -0.521, 95%CI = 0.421–0.837, *P* = 0.006) adjustment for variables. At the same time, at lower levels, there was no significant association. Vitamin E supplementation may significantly reduce the risk of BV (*p* < 0.001). In addition, the risk of having BV decreased and then increased with increasing vitamin E concentrations at high BMI levels (*p* < 0.01).

**Conclusion:**

Vitamin E at moderate to high concentrations may significantly reduce BV risk, says the study, providing clinical evidence for the prevention and the treatment of BV.

## Introduction

Females in reproductive age can be affected by BV due to an imbalance in vaginal flora [1–4]. An individual's history of sexual orientation, behavior intravaginal, the use of contraceptives, the use of antibiotics, race, education, age and menstrual cycle are all risk influences for BV [5]. The prevalence of BV varies from 20 to 60% between countries. As of 2004, the prevalence rate in the United States was 29.2%. It is noteworthy that Australia, New Zealand, and Western Europe have the lowest prevalence rates for BV [6]. BV is characterized by opportunistic bacterial overgrowth and decreased levels of lactobacilli. Approximately 90–95% of the bacteria in healthy vaginal flora are Lactobacillus [7]. BV is prone to recurrence in women receiving conventional treatment [8]. Half of women treated for bacterial vaginosis experience a recurrence within 12 months of treatment, studies have shown [9]. Recurrent bacterial vaginosis may need long-term therapy to restore the vaginal flora to a normal Lactobacillus-dominated environment. The Centers for Disease Control and Prevention recommends that bacterial vaginosis that does not recur frequently can be controlled with the same drugs used when initially infected with bacterial vaginosis [10]. The same drugs have been used for years, and many women who are affected by recurrent bacterial vaginitis cannot be treated long-term [11]. Recurrent bacterial vaginosis has a extremely destructive impact on their health and happiness, so women need effective, long-lasting treatment [12]. This underscores the need for new therapies.

Vitamin E is an essential lipid soluble nutrient that affects crucial cellular and molecular mechanisms and the regulation of gene expression and plays a vital role in various diseases. In the last 20 years, vitamin E has been demonstrated to regulate cellular responses, including survival, inflammation, migration, secretion, and immunity, through regulation of enzymes in signal transduction pathways [13]. Many in vitro/in vivo and clinical researches have been conducted with α-tocopherol, which is the most biologically active vitamin E [14]. The preventive effect of α-tocopherol in cardiovascular diseases have been reported in several cell culture and animal studies. The anti-inflammatory effects of α-tocopherol have been reported in cell culture and animal studies [15]. As mentioned above, several in vitro and in vivo investigations have confirmed the modulatory effects of vitamin E in signal transduction and inflammation [16]. In the last few years, our knowledge of vitamin E has got better significantly, and the significance of understanding the metabolomic authentication of vitamin E ramifications should not be disappreciated. The identification of key players involved in cellular and molecular mechanisms that are modified by vitamin E will undoubtedly lead to new treatment strategies for preventing disease progression [17].

The intake of several nutrients, such as vitamin B, has been reported to affect the human immune system [18, 19]. To our knowledge, no studies have assessed the association between vitamin E intake and the probability of BV prevalence. Various studies are underway to try to identify new ways to treat BV [20] This study’s purpose is to evaluate the association between vitamin E and the presence of BV. This study aimed to investigate the association between vitamin E and BV risk and to explore vitamin E supplementation as an intervention to reduce BV risk.

## Materials and methods

### Data sources and study design

**Fig. 1:**
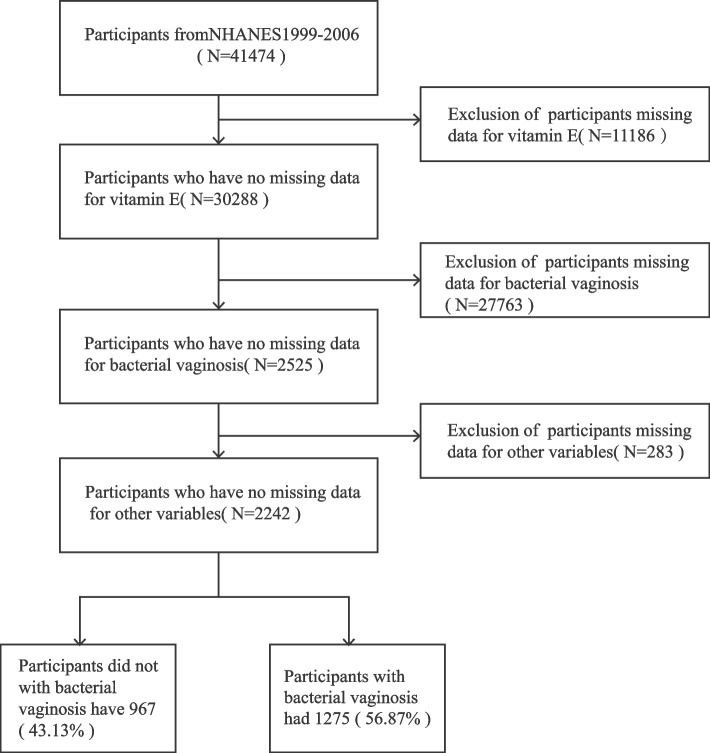
Flow chart for participant inclusion. Flow chart of participant enrollment for the analysis from the National and Nutrition Examination Survey 1999–2006 dataset

National Health and Nutrition Examination Survey (NHANES), which inquires all ages of the population’s health and nutritional status. Population statistics, health indicators, and other relevant information collected in in-home interviews provide a distinctive chance to evaluate the prevalence of BV infections in the general population, identify and ascertain risk factors, and monitor trends in BV prevalence as testing and treatment programs are established and expanded (https://www.cdc.gov/). In this study, four cycles of NHANES 1999–2006 were used, and we had 41,474 participants in America. After exclusion participants with missing variables, 2242 participants remained in this a cross-sectional study, and the flow chart of exclusion criteria is displayed in Fig. 1.

### Measurement of vitamin E

In this experiment, serum concentrations of vitamin E (α- and γ-tocopherol) were measured by high-performance liquid chromatography coupled with a photodiode array. Prior to being delivered to the National Center for Environmental Health for testing, sample vials were kept under appropriate frozen (-20 °C) storage conditions. Multiplying by 0.02, vitamin E (-tocopherol) results (ug/dL) were converted to ug/dL. Small volumes of serum (100 L) were combined with an ethanol solution, which contained two internal standards, retinol butyrate, and nonapreno-β-carotene (C45). From the aqueous phase, the micronutrients were extracted into n-hexane and dried under a vacuum. The extract was filtered after being redissolved in ethanol and acetonitrile. The filtrate was then divided equally, fed into a C18 reversed-phase column, and eluted under equal pressure by a mobile phase made up of equal parts acetonitrile and ethanol. The quantitative analysis was performed by spectrophotometry and monitored at three wavelengths, and the chromatograms were recorded. The calculations were adjusted for the difference between the peak heights of the internal standards in the calibration solution and the unknown, and α- and γ-tocopherol were compared at 300 nm. The NHANES website provides a thorough explanation of quality assurance and quality control techniques.

### Quantification of bacterial vaginitis

The NHANES Laboratory/Medical Technician Procedure Manual (LPM) includes detailed specimen collection, handling, quality control, and quality assurance instructions. After the vaginal swabs were processed, the samples were stored and transported to Magee-Women's Hospital in Pittsburgh, Pennsylvania. After Gram staining in the laboratory, BV scores were calculated using Nugent's method to quantify the mean number of lactobacilli forms in each oil-soaked area. The number of Lactobacilli spp., Gardner Ella & anaerobic GNR and Mobiluncus spp. Present was quantified. Quantitating the number of morphotypes from 0 to 4 + concerning the number of organisms present per oil immersion field and was identified as positive for bacterial vaginitis when the total bacterial count reached 7–10 (https://www.cdc.gov/nchs/nhanes/index.htm).

### Covariates

As the results of the study may be influenced by several factors, race, sex, education, age, marital status, income, alcohol intake, and poverty to income ratio (PIR) were included as covariates to make the results more accurate. Participants' race was classified as Mexican American, Other Hispanic, Non-Hispanic white, Non-Hispanic black, and Other Race; Education was categorized as below high school education level, high school education level, and above high school education; Age was equally divided into low group (14–24) and high group (25–49); Marital status has Married/Living with partner, Widowed/Divorced/Separated, Unmarried; Income includes $0–24999, $25,000–74999 and $75,000 and Over; BMI equally divided into low group (15.09–25.88) and high group (25.89–65.41); PIR was divided into three groups of similar size.

### Statistical analysis

Statistical methods such as study population description, multiple logistic regression, stratified analysis, and curve fitting were used in this study. Continuous variables were measured as "mean ± standard deviation" or "median (interquartile spacing)", and categorical variables were reported as numbers and percentages (%). To characterize participants more clearly, we analyzed the baseline characteristics of participants in the BV and non-BV populations.

In the participants' characteristics table, continuous variables were represented using weighted means (95% CI) and *P* values were measured by t-test or ANOVA (analysis of variance); categorical variables were represented using weighted percentages (95% CI) and *P* values were measured by chi-square test. The relationship between vitamin E and BV was assessed by logistic regression equations and stratified analyses. Smoothed curves were fitted using the generalised additive model (GAM), in addition to stratification for age and BMI, to explore the relationship between vitamin E and BV in different subgroups.

This study produced regression analysis tables and baseline tables of study population characteristics from two years of weighted data. The weighting analysis for the study population description was done by considering the survey design and adding in SDMVPSU in Cluster IDs, SDMVSTRA in Strata, and WTMEC2YR in Weight. The weighting analysis for the regression analysis was done by considering the survey design in Generalised Linear Models, again giving the parameters for the weighting The weighting parameters are also given. All the above data are available in the NHANES library.Stratified analysis of BV allows for knowing whether the outcome is consistently present in a given group. In this study, stratified analyses were performed for race, education level, marital status, income, alcohol intake, BMI, age, and PIR. The *p*-value results were consistent across the alcohol intake, BMI, age, and PIR strata, indicating a stable presence in that group and demonstrating that There was a significant negative association between the risk of BV and vitamin E.

The connection between vitamin E and BV was illustrated by multivariate logistic regression analysis with different models incorporating different covariates. Crude Model did not contain any covariates, Model I adjusted for race, education, and age, and Model II adjusted for marital status, income, BMI, alcohol intake, and PIR based on Model I. In this model, vitamin E was divided into four groups 41–772.5, 773–942, 942.1–1196.8, and 1198–5459 (ug/dL), with the first group serving as the reference group. We derived the relationship between vitamin E and BV in each group, performed trend analysis for vitamin E and reported the change in probability (OR) of BV for each unit increase in vitamin E. Trend analysis is a commonly used analytical method to determine the trend effect of changes in vitamin E concentration on BV by not adding adjustment variables.

To better represent the relationship between increased vitamin E intake and BV, we plotted smoothed fit curves according to Model II. In addition, we stratified age and BMI to observe the connection between vitamin E and BV in different age and BMI strata of the population.

For all analyses, the statistical significance level in this study was set at 2-sided *p* < 0.05 and 95% confidence intervals calculated. To perform data processing, the researchers used the statistical package R (The R Foundation; http://www.r-project.org; version 3.6.3) and Empower Stats software (www.empowerstats.net, X&Y solutions, Inc. Boston, Massachusetts).

### Ethics statement

Informed consent of the parents and/or legal guardians of the minors involved, all methods were conducted in accordance with relevant guidelines and regulations, ethical approval and consent to participate were obtained, and these are available at https://www.cdc.gov/rdc/.

## Result

### Participants’ characteristics

We included 2242 participants, 1275 (56.87%) were infected with BV, and 967 (43.13%) were not infected with BV. Table 1 shows the characteristics of the participants in this study. All participants, with BV compared to non-BV were more likely to be black, have lower vitamin E intake, have lower education, income from $25,000 to $74,999, higher BMI, lower PIR, higher alcohol intake, and no marital partners (all *p* < 0.05).

**Table 1:** Characteristics of 2242 women overall and by BV infection

Bacterial vaginitis			
	NO	YES	*P*-value
**Number**	1275	967	
**Age**			0.294
**Low**	29.12 (25.65,32.85)	26.57 (22.97,30.49)	
**High**	70.88 (67.15,74.35)	73.43 (69.51,77.03)	
**Race**			< 0.001
**White**	74.32 (69.39,78.70)	53.58 (46.51,60.51)	
**Black**	8.30 (6.35,10.78)	23.30 (18.50,28.91)	
**Mexican**	8.31 (6.23,11.00)	10.92 (7.63,15.39)	
**Other Hispanic**	4.68 (2.60,8.29)	6.61 (4.27,10.10)	
**Other Race**	4.39 (3.21,5.97)	5.59 (3.20,9.59)	
**Vitamin E(ug/dL)**			< 0.01
**Q1**	14.76 (11.87,18.20)	20.19 (17.34,23.39)	
**Q2**	21.43 (18.51,24.68)	25.53 (22.43,28.91)	
**Q3**	30.81 (27.88,33.91)	27.47 (23.52,31.81)	
**Q4**	32.99 (28.79,37.48)	26.80 (23.25,30.67)	
**Education**			< 0.01
< **High school**	23.11 (19.78,26.82)	26.79 (23.27,30.63)	
**High school**	19.77 (17.73,21.98)	25.33 (21.32,29.81)	
> **High school**	57.11 (53.63,60.53)	47.87 (43.89,51.89)	
**Marital status**			< 0.001
**Married/living with partner**	58.13 (54.30,61.86)	50.53 (46.13,54.91)	
**Never married**	33.58 (29.45,37.98)	33.50 (28.63,38.75)	
**Divorced/separated/never married**	8.29 (5.86,11.61)	15.97 (12.64,19.98)	
**Income**			< 0.001
**$0–24999**	21.70 (18.17,25.69)	32.45 (28.35,36.83)	
**$25,000–74999**	45.53 (40.62,50.54)	43.52 (38.66,48.50)	
**$75,000 and Over**	32.77 (27.70,38.27)	24.04 (19.83,28.81)	
**PIR**	2.97 (2.79,3.15)	2.44 (2.26,2.62)	< 0.001
**BMI**			< 0.001
**Low**	55.34 (51.67,58.95)	40.73 (34.99,46.73)	
**High**	44.66 (41.05,48.33)	59.27 (53.27,65.01)	
**Alcohol (gm)**	5.12 (3.84,6.40)	7.31 (5.73,8.90)	0.017

Mean ± SD or Median (IQR): *P* values were calculated by one-way ANOVA (normal distribution) and Kruskal‒Wallis H (skewed distribution) test % for categorical variables. *P* values were calculated by the chi-square test

### Association between vitamin E and bacterial vaginitis

Table 2 presents the weighted OR (95% CI) for BV according to vitamin E quartiles, race, etc. Logistic regression Model analysis showed that before adjustment (Crude Model), vitamin E was significantly negatively associated with BV risk in the third quartile of vitamin E. After further adjustment for race, education, age, marital status, income, BMI, alcohol intake, and PIR (Model II), Medium to high concentration of vitamin E were still significantly negatively linked to BV. In groups 3 and 4 of vitamin E concentration, there was a significant negative correlation between vitamin E concentration and BV risk (*p* < 0.05).

**Table 2:** Associations between the quartiles of vitamin E and BV infection among women

	**Crude Model**	**Model I**	**Model II**
	OR (95%Cl) *P* value	OR (95%Cl) *P* value	OR (95%Cl) *P* value
**Vitamin E (ug/dL)**			
**Q1 (41–772.5)**	Ref	Ref	Ref
**Q2 (773–942)**	-0.139 (0.659, 1.150) 0.338	-0.146 (0.643, 1.162) 0.314	-0.114 (0.671, 1.186) 0.447
**Q3 (942.1–1196.8)**	-0.429 (0.443, 0.958) 0.039	-0.476 (0.429, 0.899) 0.014	-0.396 (0.478, 0.948) 0.041
**Q4 (1198–5459)**	-0.521 (0.421, 0.837) 0.006	-0.562 (0.390, 0.832) 0.006	-0.443(0.447, 0.923) 0.032

Crude Model adjust for: none

Model I adjust for: race, education, age

Model II adjust for: race, education, age, marital status, income, BMI, alcohol, PIR

Stratified analysis was performed for age, sex, etc., as shown in Table 3. The association of vitamin E high concentration on BV was stable across alcohol intake and BMI and indicated a significant negative association (*p* < 0.05) across alcohol intake, income, marital status, education level, BMI, age, and PIR.

**Table 3:** Stratified analysis

Bacterial vaginitis					
		Vitamin E (ug/dL)			
	Participants	Q1	Q2	Q3	Q4
**Age**		Ref			
**Low**	1084	Ref	0.942 (0.696, 1.275) 0.6979	0.767 (0.532, 1.106) 0.1557	0.603 (0.378, 0.964) 0.0344
**High**	1158	Ref	1.126 (0.692, 1.830) 0.6336	0.799 (0.504, 1.268) 0.3411	0.753 (0.475, 1.191) 0.2251
**Race**					
**White**	905	Ref	0.938 (0.590, 1.492) 0.7876	0.674 (0.416, 1.092) 0.1091	0.698 (0.432, 1.129) 0.1425
**Black**	592	Ref	0.884 (0.575, 1.359) 0.5747	0.704 (0.423, 1.172) 0.1777	0.446 (0.242, 0.824) 0.0099
**Mexican**	577	Ref	1.008 (0.619, 1.640) 0.9750	0.708 (0.426, 1.178) 0.1842	0.699 (0.407, 1.203) 0.1959
**Other Hispanic**	85	Ref	0.970 (0.227, 4.144) 0.9672	0.720 (0.159, 3.260) 0.6696	0.619 (0.117, 3.274) 0.5722
**Other race**	83	Ref	2.377 (0.524, 10.792) 0.2619	8.441 (1.083, 65.773) 0.0417	1.352 (0.231, 7.934) 0.7380
**Education**		Ref			
< **High school**	983	Ref	0.972 (0.702, 1.346) 0.8637	0.767 (0.515, 1.142) 0.1914	0.645 (0.405, 1.025) 0.0636
**High school**	422	Ref	1.120 (0.591, 2.124) 0.7276	0.727 (0.394, 1.341) 0.3070	0.348 (0.183, 0.662) 0.0013
> **High school**	837	Ref	0.920 (0.546, 1.550) 0.7544	0.757 (0.454, 1.263) 0.2871	0.941 (0.562, 1.578) 0.8185
**Marital status**		Ref			
**Married/living with partner**	949	Ref	0.639 (0.387, 1.055) 0.0799	0.653 (0.409, 1.043) 0.0746	0.628 (0.395, 0.997) 0.0488
**Never married**	1102	Ref	1.023 (0.751, 1.392) 0.8863	0.799 (0.549, 1.162) 0.2401	0.585 (0.362, 0.944) 0.0281
**Divorced/separated/never married**	191	Ref	2.438 (0.694, 8.559) 0.1643	0.420 (0.129, 1.369) 0.1502	0.400 (0.121, 1.326) 0.1340
**Income**		Ref			
**$0–24999**	746	Ref	0.974 (0.652, 1.457) 0.8995	0.707 (0.457, 1.095) 0.1202	0.711 (0.436, 1.160) 0.1717
**$25,000–74999**	971	Ref	1.066 (0.717, 1.585) 0.7533	0.599 (0.389, 0.922) 0.0199	0.529 (0.339, 0.825) 0.0050
**$75,000 and Over**	525	Ref	0.787 (0.444, 1.397) 0.4135	0.948 (0.520, 1.731) 0.8628	0.713 (0.375, 1.357) 0.3030
**PIR**		Ref			
**0—1.19**	747	Ref	0.904 (0.606, 1.347) 0.6197	0.588 (0.384, 0.901) 0.0148	0.504 (0.305, 0.833) 0.0076
**1.2—3.13**	742	Ref	1.069 (0.685, 1.669) 0.7689	0.750 (0.458, 1.229) 0.2542	0.669 (0.406, 1.104) 0.1158
**3.14—5**	753	Ref	1.041 (0.634, 1.709) 0.8736	0.975 (0.578, 1.645) 0.9240	0.786 (0.460, 1.343) 0.3788
**BMI**		Ref			
**Low**	1121	Ref	0.782 (0.559, 1.095) 0.1520	0.652 (0.441, 0.963) 0.0315	0.636 (0.409, 0.989) 0.0445
**High**	1121	Ref	1.344 (0.915, 1.973) 0.1315	0.795 (0.545, 1.162) 0.2361	0.671 (0.456, 0.989) 0.0435
**Alcohol (gm)**	2242	Ref	0.973 (0.759, 1.248) 0.8320	0.708 (0.541, 0.926) 0.0118	0.625 (0.470, 0.831) 0.0012

The adjusted model in the stratification analysis was constructed based on Model II, adjusted for race, sex, education, age, marital status, income, BMI, alcohol and PIR. Stratified variables were excluded from the adjusted model

**Fig. 2:**
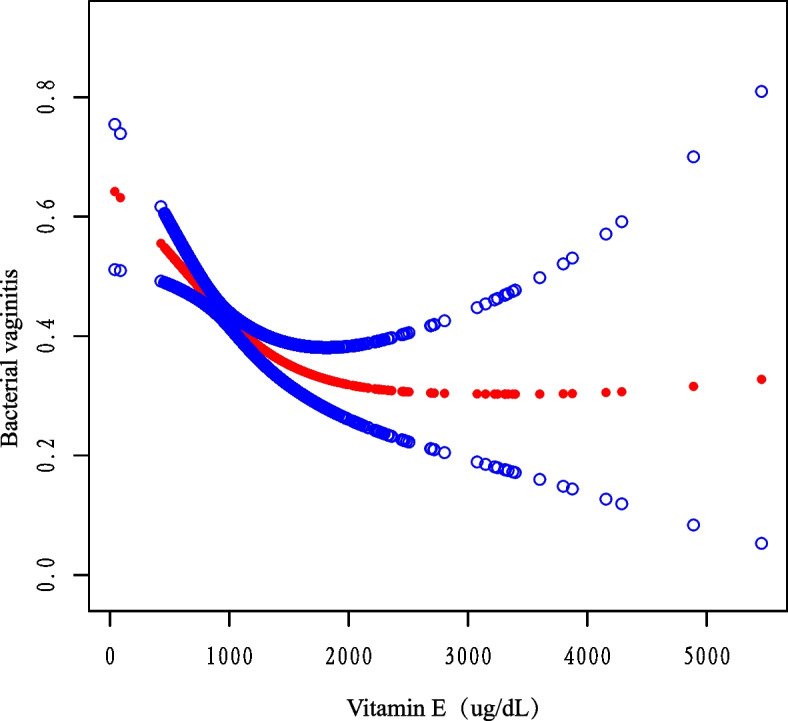
Correlation between Vitamin E and Bacterial vaginitis. The natural spline curve shows a linear relationship between vitamin E and BV (*P*_linearity_ < 0.001). The area between the blue dashed lines is considered to be the 95% confidential interval. Each red dot reveals the concentration of vitamin E, forming a continuous fitted curve. Ratios are based on model II in a multivariate logistic regression model, adjusted for all confounders (race, sex, education, age, marital status, income, BMI, alcohol, PIR)

For vitamin E and BV, the relationship between the curves is shown in Fig. 2, illustrating that the connection between vitamin E and BV is nonlinear in the curve fit, from which it can be seen that as the concentration of vitamin E increases, the risk of acquiring BV decreases gradually and then increases slightly (*p* < 0.001).

**Fig. 3:**
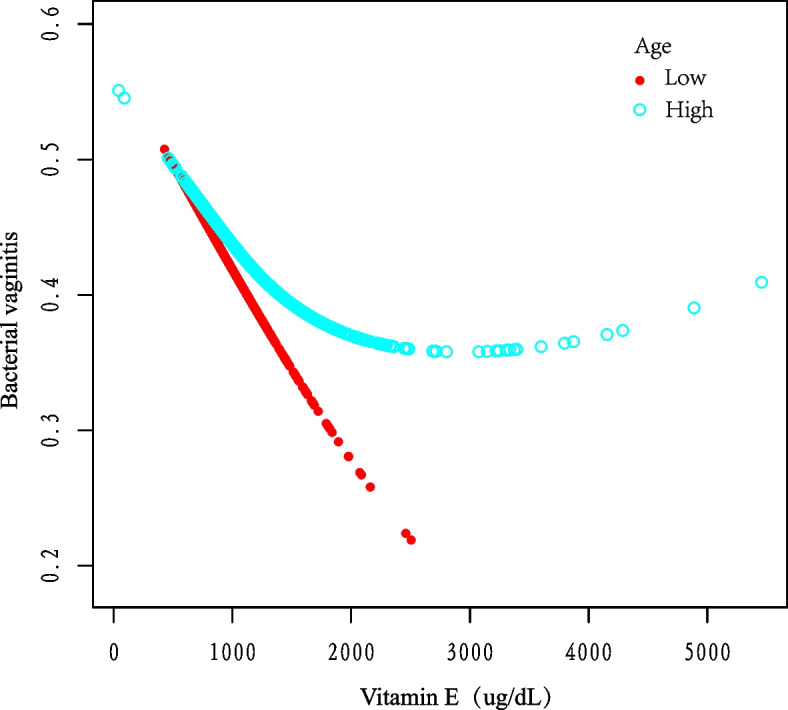
Correlation between vitamin E and BV infection stratified by age. Fitting curve of the association between vitamin E and BV infection, stratified by age, with age = 24 taken as the dividing criterion. Race, sex, education, marital status, income, BMI, alcohol and PIR were adjusted

Figure 3 Stratifies age and shows the curves of vitamin E to BV. The hazard of acquiring BV showed to reduce with increasing vitamin E concentrations when age was in the Low group (*p* < 0.01).

**Fig. 4:**
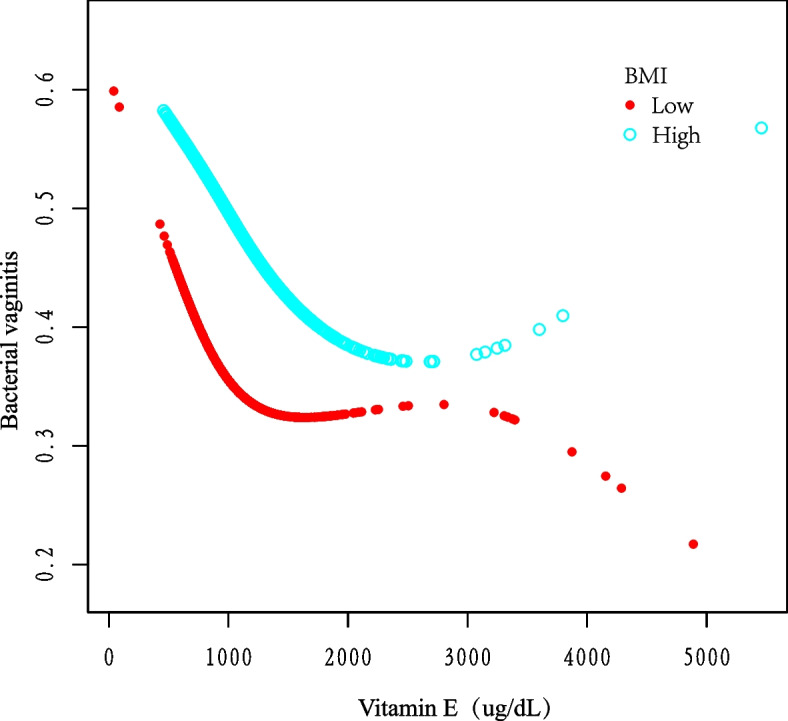
Correlation between vitamin E and BV infection stratified by BMI. Fitting curve of the association between vitamin E and BV infection, stratified by BMI, with BMI = 25.88 taken as the dividing criterion. Race, sex, education, age, marital status, income, alcohol and PIR were adjusted

Figure 4 Stratifies BMI, and when BMI was in the High group, with increasing vitamin E concentrations, the risk of having BV decreased when the concentration was before 1841 ug/dL and increased when the concentration was greater than 1841 ug/dL (*p* < 0.01). Among them, the change in concentration before 1841 was significant (*p* < 0.01).

## Discussion

Using multiple regression analysis with vitamin E as an exposure factor in quartiles, we found a significant negative association with the risk of BV at vitamin E concentrations in the third and four quartiles. After adjusting for variables multiple times, we found a correlation between a higher probability of BV prevalence and lower vitamin E intake.

The characteristic of BV is the overgrowth of vaginal anaerobic bacteria, which results in vaginal itching and unpleasant fishy-smelling discharge accompanied by an increase in vaginal pH [21, 22]. The prevalence of BV is closely related to the strength of the body's immune function, and it has been shown that vitamin E can reduce the prevalence of BV by improving the body's immune function [23].

Vitamin E, as a potent antioxidant, has a relationship with the immune response. Studies have shown that vitamin E has some anti-inflammatory effects [24]. When attacked by bacteria, leukocytes produce a large number of free radicals to resist foreign bacteria, and the metabolism of free radicals in the body is abnormal. At the same time, the body produces highly reactive free radicals in the cellular respiratory chain during normal metabolism, especially ROS radicals [25]. ROS can damage cellular components, such as DNA and membrane lipids [26]. A certain amount of ROS helps lymphocytes participate in the immune response [27], but high ROS can cause apoptosis or necrosis [28]. In response to oxidative toxicity, cells preclude or neutralize the ROS produced in the metabolism by various antioxidant mechanisms. When the produce of ROS outstrips the cellular antioxidant scavenging capacity, bacterial vaginitis takes place [29]. In contrast, vitamin E, as a fat-soluble antioxidant, can scavenge excess free radicals in the body and maintain stable ROS levels, as well as regulate signal transduction and gene expression in immune system diseases and may play a role in BV prevention. In conclusion, by improving immune function through antioxidant and anti-inflammatory effects, vitamin E may be able to reduce the risk of BV.

As we know, this cross-sectional study is the first one to analyze the relationship between BV prevalence probability and vitamin E intake in US women by age and BMI subgroups. There is only one study on the relationship between dietary nutrient intake and BV that mentions a negative correlation between vitamin E and severe BV, but it was not analyzed specifically [23]. Notably, in Fig. 3, it can be concluded that vitamin E supplementation may significantly reduce the risk of BV in young women aged 14–25 years. Furthermore, in our stratified analysis of BMI (Fig. 4), we found that the risk of having BV decreased and then increased with increasing vitamin E concentrations at high BMI levels. Similar to our findings, Agarwal, S et al. used total nutrient intake data from the National Health NHANES 2001–2008 to compare the micronutrient intake status of overweight and obese populations with those of normal weight and found that the prevalence of malnutrition was higher in obese populations and that vitamin E intake did not meet the normal needs of the organism [30].

In summary, we conclude that moderate to high concentrations of vitamin E can significantly reduce the prevalence of BV, and this result is expected to provide clues and etiological hypotheses for the investigation of the developmental mechanisms of BV, as well as provide effective feedback for the prevention and treatment of BV.

Despite the promising results, there are still problems. Firstly, as it is a cross-sectional study, the causality of exposure and outcome is not sure. Even with multiple variable adjustments, there are still some potential unmeasurable confounders present that introduce errors in the findings. Second, due to a lack of data support, current US clinical guidelines do not recommend screening for BV in asymptomatic pregnant women [31, 32], the BV prevalence levels in the NHANES database may below the actual one.

In the present investigation, although we derived evidence for a correlation between vitamin E and BV, its specific physiological mechanisms need to be further investigated. To optimize this study and provide new ideas for future studies, we propose several suggestions: First, to construct a risk assessment tool for BV disease based on Amsel criteria to identify high-risk groups for BV and improve the efficiency of BV prevention. What’s more, further survey is exceedingly needed to clarify the mechanism how vitamin E reduces the prevalence of BV, to find BV-related biomarkers and promote the development of new drug targets.

## Conclusion

In conclusion, when serum vitamin E was at moderate to high concentrations, it was significantly associated with a reduced prevalence of bacterial vaginal infections. Moreover, our study found that those infected with BV were more likely to have lower vitamin E intake, higher BMI, higher alcohol intake, and no marital partners. This study provides some data to support the clinical treatment and prevention of BV. However, more evidence is needed from randomized controlled trials.

## Data Availability

Detailed data available at https://www.cdc.gov/nchs/nhanes/index.htm

## Abbreviations

BV: Bacterial vaginitis
NHANES: National Health and Nutrition Examination Survey
IQR: Interquartile range
SD: Standard deviation
PIR: Poverty Income Ratio
BMI: Body Mass Index
CI: Confidence Interval
